# Surface-Based Display of Volume-Averaged Cerebellar Imaging Data

**DOI:** 10.1371/journal.pone.0133402

**Published:** 2015-07-31

**Authors:** Jörn Diedrichsen, Ewa Zotow

**Affiliations:** Institute of Cognitive Neuroscience, University College London, London, United Kingdom; Tokyo Medical and Dental University, JAPAN

## Abstract

The paper presents a flat representation of the human cerebellum, useful for visualizing functional imaging data after volume-based normalization and averaging across subjects. Instead of reconstructing individual cerebellar surfaces, the method uses a white- and grey-matter surface defined on volume-averaged anatomical data. Functional data can be projected along the lines of corresponding vertices on the two surfaces. The flat representation is optimized to yield a roughly proportional relationship between the surface area of the 2D-representation and the volume of the underlying cerebellar grey matter. The map allows users to visualize the activation state of the complete cerebellar grey matter in one concise view, equally revealing both the anterior-posterior (lobular) and medial-lateral organization. As examples, published data on resting-state networks and task-related activity are presented on the flatmap. The software and maps are freely available and compatible with most major neuroimaging packages.

## Introduction

Surface-based analysis and visualization methods have greatly contributed to our understanding of the functional organization of the neocortex [1–3]. Traditional volume-based displays provide only a particular view of the data, and even adjacent slices through the volume can often tell a very different visual story. Inflated representations of the cortical sheet allow for the visualization of functional activity patterns across the entire neocortex. Surface-based methods also allow for improved accuracy of inter-subject alignment, accounting for individual variability in the structure of cortical folding [4–8].

While surface-based analysis and visualization have become a gold standard in many laboratories and software packages, they are not yet used for data from the cerebellum. There are two reasons for this. First, the cerebellum is much more tightly folded than the human neocortex, with individual folia being 1-2mm wide [9] and the complete cerebellar cortical sheet approximately 1.5-2m long [10]. Complete unfolding of the cerebellar cortex based on MRI scans requires a resolution higher than 200μm [11], which is typically not available in human imaging studies. Secondly, for the currently typical resolution of functional imaging data (2-4mm), the activation from adjacent folia is averaged. Therefore, even small and focal activations cannot unambiguously be assigned to a specific folium, reducing the precision that could be gained by reconstructing individual surfaces.

Thus, instead of developing a surface-based normalization for individually reconstructed cerebellar surfaces, the aim of this paper is to provide a flat representation of the cerebellum as a visualization tool for volume-averaged cerebellar data. Volume-based alignment of cerebellar data has improved greatly through the development of non-linear morphing algorithms and high-resolution non-linear templates [12–15]. These methods superimpose individual cerebellar lobules with good precision [16], even if they cannot bring individual folia into alignment.

**Fig 1:**
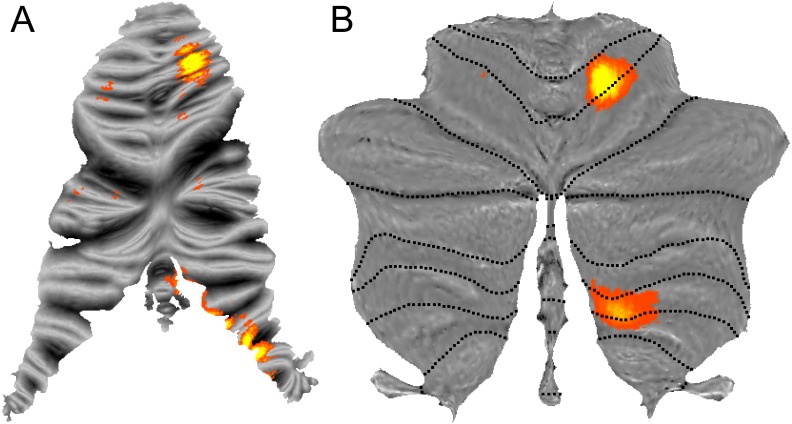
Representation of individual fingers in the human cerebellum. Shown is the classification accuracy with which the moved finger can be determined from the local pattern of activity, with a threshold of *z*>1 [17]. (**A**) Data projected onto a surface based on a single anatomy [2] displays single closed activation clusters as a fractured series of blobs. (**B**) Projection to the new flatmap ensures that single clusters in the volume are also presented as such on the surface.

A reconstruction of an individual cerebellar surface has been published previously [2]. This map reflects the folding of the cerebellum to the level of groups of folia of a single individual, as accurately as is possible for an anatomical scan with a 1mm resolution. For display of functional group data, however, this surface is unsatisfactory. For example, in the volume-based analysis, there are two sites of individual finger representations in the human cerebellum [17]. However, when projecting this data onto the aforementioned flatmap, both loci are fractionated and distributed over a number of folds of the surface (Fig 1a). This problem arises from the fact that the level of detail of the anatomical unfolding is too high for the resolution of volume-averaged group data.

Therefore, we aimed here to develop a surface representation of the body of cerebellar grey matter that meets the following criteria: It should show data on the level of lobules, but not attempt to unfold the cerebellar surface down to the level of individual folia. The projection should ensure that closed clusters in the volume are also displayed as closed clusters on the surface (Fig 1b). The projection method should ensure that the entire data from cerebellar grey matter is represented on the surface. Lastly, the 2D-map should provide a truthful representation of the size of different cerebellar structures with the surface area corresponding approximately to the displayed volume.

After presenting and evaluating the surface representation, we demonstrate the utility of the flatmap as a tool for visualizing various kinds of cerebellar data. We first project the probabilistic atlas of the lobular structure [12] to the flatmap, which provides a first basic anatomical orientation. We then present maps of functional resting-state connectivity obtained from a large data set (N = 1000) [18], which reveal the intricate pattern of connectivity with the neocortex. Finally, we present task-based activation maps using a subsample (N = 100) of participants from the Human Connectome Project (HCP) [19], who were scanned on a variety of functional tasks. While the results from these data sets have been analyzed and published for the neocortex [20], the authors did not present the cerebellar findings in full detail. Together, the presented data sets should provide a useful reference relative to which new results can be interpreted.

## Method

### Data and ethics statement

Anatomical and functional data from five published studies was utilized [12, 16–18, 20]. All experimental procedures were approved by the respective local ethics committees, as documented in the original papers. The re-analysis of these non-sensitive and fully anonymized data did not require separate approval under the rules of the University College London research ethics committee.

### Volume preparation

The flatmap was based on the anatomical data from 20 participants originally used for the generation of the spatially unbiased infratentorial template (SUIT) [12]. The scans were segmented into grey and white matter using unified segmentation [13] and the cerebellum was isolated from the neocortex using an automatic algorithm [12]. After the first affine alignment to the SUIT template, we utilized the fast diffeomorphic anatomical registration algorithm (Dartel) [14] to generate a new, slightly sharper average grey-matter and white-matter template.

The extent of the cerebellar white-matter body was then estimated applying a threshold of *p*>0.4 to the average map of white-matter probabilities. The remaining cerebellar voxels were labeled as grey matter if the grey matter probability exceeded 0.5. The maps were then hand-edited to ensure that the grey matter of the posterior vermis was separated from the abutting hemispheres of lobule HIX.

### Surface reconstruction

**Fig 2:**
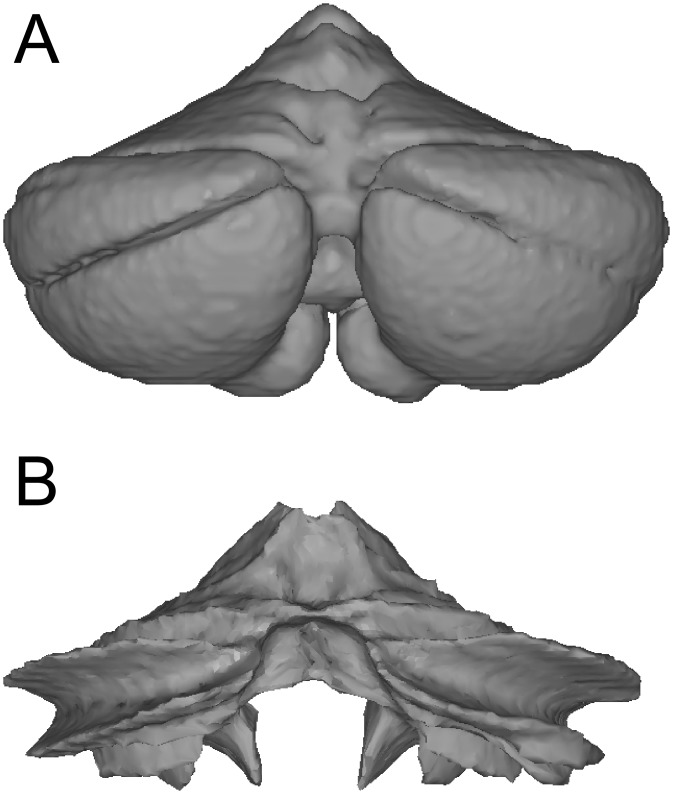
Surfaces defined on a group-averaged anatomical template image of the human cerebellum. (**A**) The outer surface constitutes a hull around the average grey matter body. (**B**) The inner surface is placed on the boundary between average white and grey masses.

We reconstructed the outer grey-matter surface (Fig 2a) and the inner white-matter surface (Fig 2b) using the Freesurfer packages [1]. After smoothing and topology correction, both surfaces were inflated. Twenty-two pairs of reference points were then selected, which defined an approximate mapping between the vertices of the two surfaces. These points were placed on clearly defined landmarks, such as the base and superficial aspect of major fissures. Each vertex from the inflated outer surface was then projected to the inflated inner surface, using the connection lines between the predefined vertex pairs as guides. We then resampled the inner surface into the grid of the outer surface. This resulted in two surfaces on which the corresponding vertices reflected the deeper and the more superficial parts of the same lobules.

### Flattening & distortion assessment

**Fig 3:**
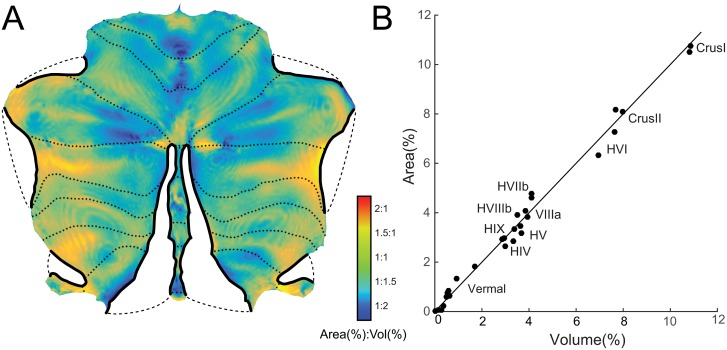
Distortion of the flatmap in representing cerebellar grey-matter volume as surface area. (**A**) Flatmap with superimposed distortion factor (ratio of Area to Volume). Orange / red areas indicate regions that are disproportionally large on the flatmap, turquoise / blue areas indicate regions that are disproportionally small. Dotted lines indicate boundaries between lobules. Thick black lines on the perimeter indicate where cuts have been made to the map. The areas connected with dashed lines are immediately adjacent in the volume, but are unfolded in the flatmap to minimize distortion. (**B**) Volume of each lobule (in % of total grey-matter volume) plotted against the corresponding area on the flatmap (in % of total map area). Plotted are 28 compartments, hemisphere and vermis of each of the main lobules, as defined in the probabilistic atlas of the human cerebellum.

We then applied cuts to the surface to enable flattening. After removing the cerebellar peduncles from the surface, we inserted cuts in the horizontal fissure, the superior-posterior fissure, between the posterior vermis and lobules HVII—HIX, and between the HX and HVIII (thick black lines in Fig 3a). The inflated grey-matter surface was then transformed into a flat representation using Caret software [3].

The outer boundary and overall layout of the flatmap was initially adjusted by hand to ensure a roughly symmetric layout and a representative size of the lobules. With the overall outline fixed, we then applied a custom-written Matlab algorithm, which moved all interior vertices to minimize the distortion of the map. Specifically, the goal was that the area of triangle was proportional to the grey matter volume that it represented.

### Functional volume to surface mapping

**Fig 4:**
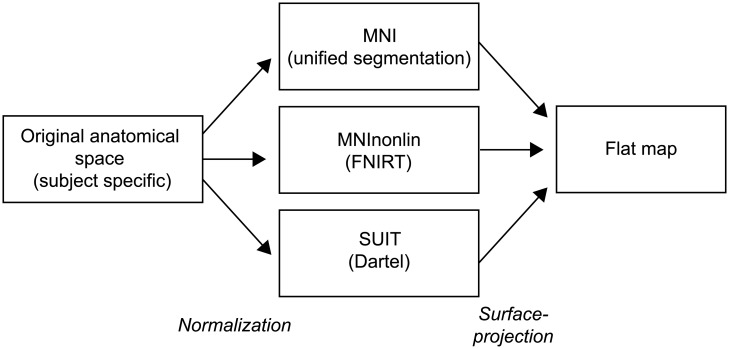
Surface-based mapping pipeline for cerebellar data. Functional data is first normalized using standard volume-based methods and then projected onto the flat representations using corresponding vertices on outer and inner surface. For the process of surface projection, it is therefore important to take into account the type of volume-based normalization algorithm used.

The surfaces are designed to display functional data that has been normalized into a volumetric group atlas space. Non-linear normalization algorithms [14, 15, 21] usually yield the highest accuracy of volumetric alignment, especially when they are combined with a non-linearly generated template that preserves the anatomical details [12]. For cerebellar data, SUIT normalization using Dartel [12], unified segmentation and normalization, as implemented in the statistical parameter mapping (SPM) toolbox [13, 22], and the fast nonlinear image registration (FNIRT) to the MNI152 template as implemented in the FMRIB software library (FSL) [21], are currently the best available and most commonly-used normalization methods of this kind (4). Although all are approximately unbiased relative to each other [16], they do slightly differ. We therefore morphed the surfaces generated in SUIT space into the space defined by the other two methods, such that volumetric group data using either approach can be easily mapped onto the same surface (Fig 4). For accurate mapping results, it is therefore important to specify the algorithm that was used for the volume-based normalization correctly.

Volumetric data can be mapped to the atlas surface by sampling the 3D image along the line connecting the two corresponding vertices on the outer and inner surface. The spacing of the sampling and the range of sampling depth can be adjusted. This mapping procedure has the advantage that it ensures a veridical and representative representation of the volumetric data on the surface. Only the value coming from grey matter voxels will be displayed, and each grey matter voxel is represented through similarly sized patch on the flatmap.

The sampled voxels then need to be integrated to assign a value to each vertex, with different types of functional data requiring different ways of integration. Most typically, the mean of the voxels underlying the vertex is used, such as in the example of Fig 1. However, in the case of discrete labels, such as the resting-state networks, the mode (the most frequently occurring value) is more appropriate. Finally, for statistical maps, a glass-brain projection, which displays the maximum value of the underlying voxels, may be desirable to ensure that all significant clusters are visible on the surface. For details on mapping procedures, see below.

### Probabilistic atlas

The probabilistic atlas of cerebellar anatomy [16] is based on the hand-segmentation of the lobular compartments in 20 healthy participants, separating cerebellar lobules I-IV, V, VI, VIIa (Crus I and Crus II), VIIb, VIIIa, VIIIb, IX and X. For all lobules, the left and right hemispheres are labeled as separate compartments. For the posterior cerebellum (lobules VI-X), for which a clear anatomical boundary between vermis and hemisphere can be found, the atlas also defines a separate compartment for the vermal aspect.

To display the atlas on the surface, we first projected the probability maps for each of the compartments onto the vertices, averaging probabilities across the different sampling depth. We then generated a winner-take-all map, by assigning each vertex to the compartment with the highest average probability.

### Functional resting-state connectivity

As one example for the utility of the surface for the visualization volumetric group data, we used a map of resting-state connectivity with the neocortex [18]. This map is based on a parcellation of the neocortex into 17 networks according to the correlation between low-frequency fluctuations in the blood-oxygenation dependent (BOLD) signal [23]. Each cerebellar voxel was then assigned to the cortical network with which it correlated most.

The map of voxel assignments was projected onto the flat surface by assigning each surface vertex the most-often occurring label value for the underlying voxels (the mode across all sampled values).

### Task-based activity maps

To demonstrate the use of the surface for the display of task-based activation, we used the openly available neuroimaging data from the Human Connectome Project (HCP, [19]). We randomly selected 100 healthy, unrelated subjects who completed the entire fMRI protocol. Each subject performed seven tasks covering a number of different domains (motor, working memory, language, and emotion, social, relational and reward processing), which are described in detail in [20].

We used the results from the first-level general linear model (GLM) analysis which was pre-processed using the HCP pipeline, aligned to MNI space using FNIRT [24], and finally volume-smoothed at 4mm full-width at half maximum (FWHM).

We submitted the activation values for each participant to a second-level (random-effects) group analysis. At each cerebellar voxel, we performed a *t*-test comparing a given task to its control condition. The statistical threshold was adjusted to control the family-wise error (FWE) across the volume of the cerebellar grey matter, using Gaussian-field theory, as implemented in SPM [25]. This led to an adjusted threshold of *t*_(99)_ > 5.473, *p*<0.001, *pFWE* = 0.05. The volume-based results were then mapped onto the surface that was specifically morphed to fit the non-linear template in FSL.

To display the statistical values on the flatmap, we used a ‘glass brain’ projection—assigning each vertex with either the minimum or maximum statistical value of the underlying voxels—whichever had the higher absolute value. While this approach over-emphasizes the areas of significance, it has the distinct advantage that it shows all areas of significant differences that are detected in the volume.

Finally, we used the tasks that involved movements of different body parts to generate a rough somatotopic map on the surface. For each movement condition (left foot, right foot, left hand, right hand, tongue), we first averaged the activity values across participants and then across the voxels for each vertex. Each vertex was then assigned to the body part with the maximal activity and values above an arbitrary threshold level (>25) were displayed on the flatmap.

### Software Toolbox

The maps and software are part of the SUIT toolbox [26], which is distributed under the Creative Commons Attribution-Noncommercial Unported License. It can be freely used for non-commercial purposes, as long as proper attribution in form of acknowledgments and links (for online use) or citations (in talks and publications) is given. The software for mapping and display is written in Matlab (The MathWorks, Inc., Natick, MA) and requires SPM12 [22] to be installed. The maps are created to also work with the freely available surface-based display software Caret [3].

## Results

### Flatmap and area distortion

The resultant flatmap is shown in Fig 3a. The anterior lobe is represented on the upper part of the map, and the “wings” are formed by the hemispheres of lobule VIIa (Crus I and II). The posterior vermis is separated from the hemispheres through a cut and therefore protrudes as a “tail”. Equally, two lateral cuts were inserted between lobule HVI and Crus I, and between Crus I and II (indicated by thick black lines, with dashed lines linking immediately adjacent areas). Finally, lobule HX was separated from HVIII, even though it directly abuts this structure in the volume.

The flatmap was generated such that the size of each area on the map is proportional to the grey matter volume it represents. To assess the deviation from this ideal, we computed for each tile the percentage of the total map area it occupied and the percentage of total grey-matter volume it represented. We then visualized the log of the ratio of these two numbers—which ideally should be zero—as a color map on the surface (Fig 3a). Tiles that are too small for the underlying volume are shown in blue. This occurs mostly in areas where the distance between the inner and outer surface is large, such as in the medial portion of Crus I and II. Areas that are too large for the volume they represent are shown in red. These areas are found in places where the distance between the grey- and white-matter surfaces is small (in the center of a lobule) and around the fringes of the map. The latter could be remedied by applying more small cuts around the perimeter of the map. This, however, would lead to more discontinuities in the projection—that is, a single activation blob could be represented in two disconnected areas of the map.

Most vertices, 93.1%, showed distortions (area-to-volume ratios) between 1:2 and 2:1, and 75.8% showed a distortion between 1:1.5 and 1.5:1. The relative distortion was relatively balanced across the different lobules. To assess this, we assigned each vertex to one of the 28 lobular compartments defined in a probabilistic volume-based atlas (see methods) [16]. Equally, we also assigned each voxel of the volume-based atlas to the compartment with the highest probability, thereby estimating the average volume of each lobule. We then plotted the area for each compartment on the flatmap against the volume occupied in the volume-based atlas (Fig 3b).

The achieved and desired areas were in close agreement—the correlation was *r* = 0.994. The largest deviation was in the vermal lobule VIIb, which was overrepresented by factor 2 in the flat representation. This is due to the distortion that occurs at the end of the vermal cut. However, given the overall relatively small size of this area, the deviation can be considered minor.

Thus, while not perfect, the map constitutes a practical compromise between a veridical representation of the whole cerebellar volume, and the requirement to project a complex 3-dimensional structure to a surface without too many discontinuities (cuts).

### Probabilistic atlas

One basic and important aspect of cerebellar organization is the anterior-to-posterior division into lobules [27]. Because the boundaries between lobules, the cerebellar fissures, are easily recognized based on the gross anatomy, the cerebellar imaging literature has focused on functional differences between these lobules. Based on the notation introduced by Larsell, the lobules are denoted by roman numerals from I to X [28, 29] with a prepended H to distinguish the hemispheric from the vermal compartment [27]. In the human brain, lobules I-III are very small. Furthermore, the largest lobule is HVII, which is further subdivided into HVIIa (Crus I and Crus II) and HVIIb. Similarly, lobule HVIII is divided into two parts, separated by the intrabiventer fissure [27]. Here, we are using a probabilistic atlas of the lobular organization after alignment to the SUIT template [16]. In this atlas the left and right hemispheres are labeled as separate compartments. For lobules VI-X, for which a clear anatomical boundary between vermis and hemisphere can be found, the atlas also defines a separate compartment for the vermal aspect.

**Fig 5:**
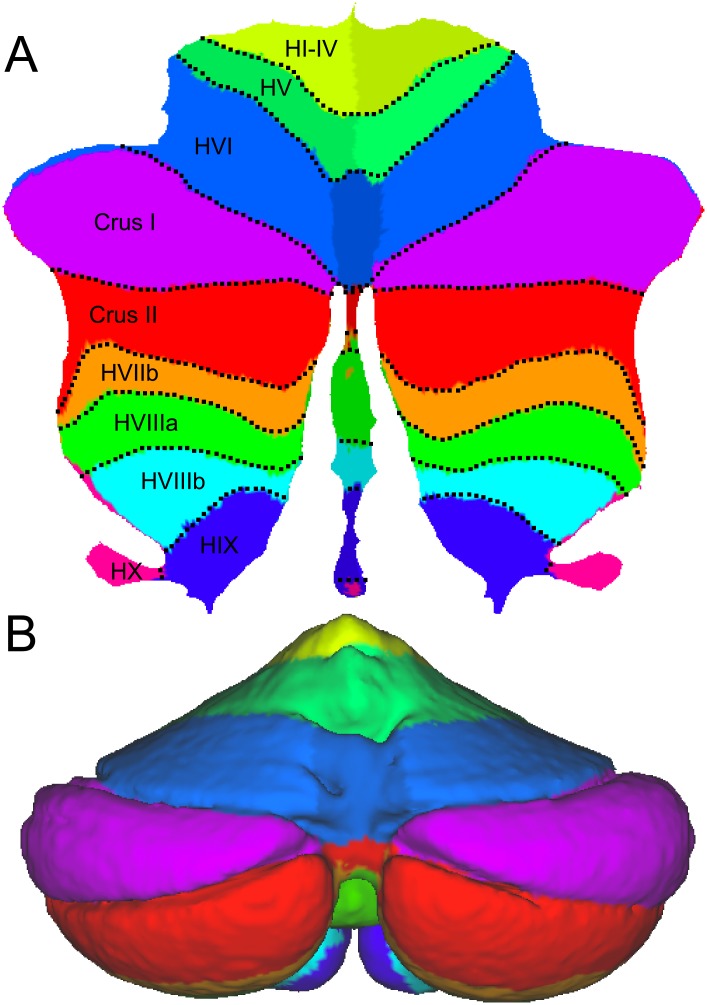
Probabilistic atlas of the cerebellar lobules. (**A**) The compartments of the cerebellar atlas [16] projected to the flatmap. Note that for lobule VI-X, a vermal and two hemispheric compartments (shown in slightly different colors) are defined. (**B**) The same data displayed on a posterior view of the outer surface.

The probability maps for each of the compartment were mapped to the surface and each vertex was then labeled with the color of the compartment with the maximum probability (Fig 5a and 5b). The representation of the probabilistic atlas on the surface shows clearly the relationship between different lobular sizes—especially emphasizing that nearly half of the cerebellar gray matter is contained in lobule HVII [30].

**Fig 6:**
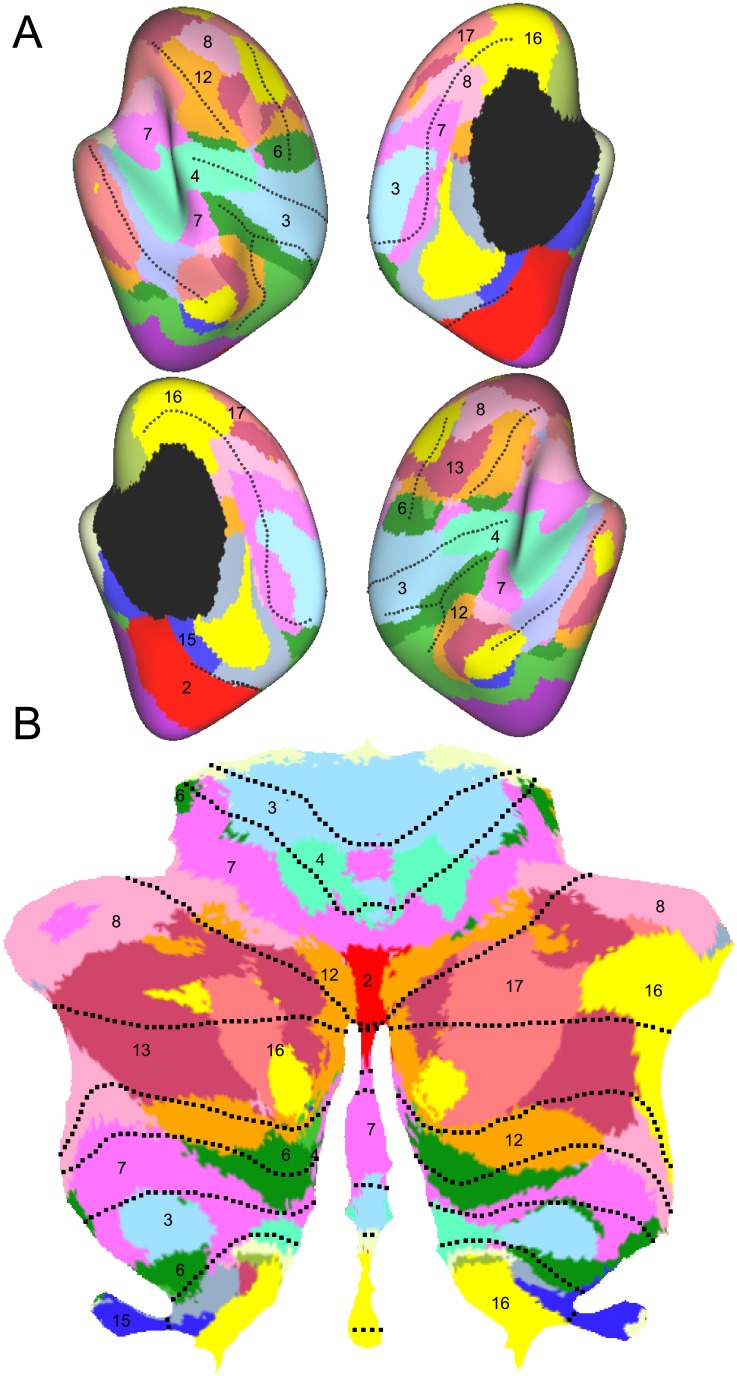
Atlas of cerebellar-cortical connectivity. (**A**) Cortical networks of resting-state connectivity [23]. 17 networks are shown on an inflated cortical surface of the left and right hemisphere—with both the lateral and medial surface shown. (**B**) Map showing the cortical resting-state network that correlated best with the activity in the corresponding cerebellar area [18]. Maps are based on N = 1000 subjects.

**Fig 7:**
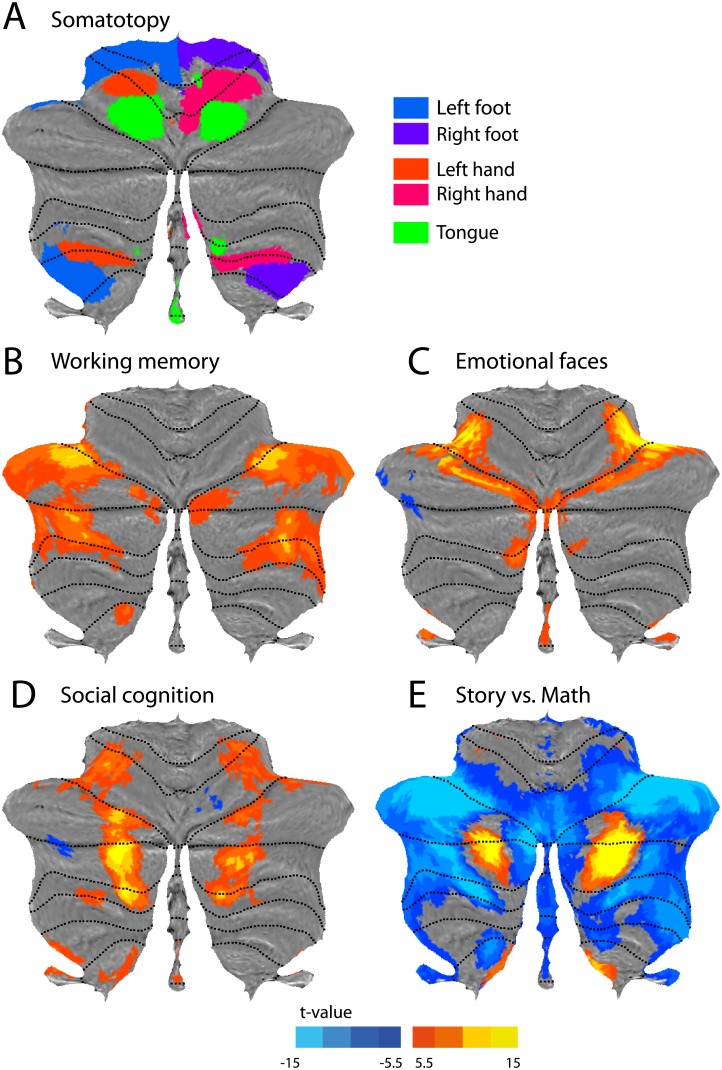
Functional activity maps from the Human Connectome Project. (**A**) Sensorimotor topography of activation for hand, foot and tongue movements. (**B**) Working memory; contrast between a 2-back and 0-back condition. (**C**) Emotion processing; contrast between matching emotional faces vs. matching neutral shapes. (**D**) Social cognition; observing dot motion with intentional content vs. random dot motion. (**E**) Language vs. mathematical processing. Positive values indicate higher activity during processing of a story vs. arithmetic operations. Negative values represent the opposite contrast. All maps are based on N = 100 subjects. All colored areas in cognitive maps (B-E) exceed an FWE-corrected significance threshold of *p*<0.05.

For easy reference, the boundaries between the lobular compartments can be superimposed on other functional maps as dotted lines (see Figs 5–7). However, it should be kept in mind that the location of the boundaries is associated with some uncertainty. First, even the best current volume-based normalization algorithms still do not lead to a perfect overlap of the lobules [16]. Furthermore, the mapping to a flat surface yields further inaccuracies. Although we did our best to ensure that the corresponding vertices on inner and outer surface belonged to the same lobule, the assignment may vary slightly with the relative depth at which the information is sampled. Because one will typically present the signal averaged across a number of depths, the vertices close to the boundaries between lobules will usually mix some information across different lobules.

### Resting-state connectivity maps

To investigate the patterns of cerebellar-cortical connectivity, Buckner et al. [18] studied the correlations in spontaneous BOLD fluctuations during resting state scans. First, the neocortical surface was divided into 17 different networks using independent component analysis (Fig 6a) [23]. Each cerebellar voxel could then be assigned to the cortical network with which it showed the highest correlation.

The patterns of cerebellar-cortical connectivity as presented on the flat representation (Fig 6b) provide a number of important insights. First, there is a repeating representation of cortical areas across the cerebellum [18]. Clearly visible are the motor networks (3,4) in the anterior (HV-HVI) and the posterior cerebellum (HVIII), both of which each surrounded by the premotor networks (6,7). Lobule HVII is occupied by a series of prefrontal and parietal networks, some of which are again represented in lobule HIX.

Second, the display of the resting state networks on the flat representation makes imminently clear that the boundaries of the resting-state networks do not respect lobular borders. For example, the motor network lie right on the boundary between lobules HV and HVI, and the prefrontal networks span Crus I, II, and lobule HVIIb. This is not an artifact of the flat representation, but also is clearly visible in the volume-based maps. It indicates that most cerebellar fissures do not necessarily coincide with functional boundaries. The flat representation also makes clear that the functional organization varies considerably according to the medial-to-lateral position on the cerebellar surface. For example, in lobules HVII, the networks that are located more laterally in the cerebellum encompass generally more rostral areas in the prefrontal cortex. Thus, it is possible that lobules HVII mirror the caudal to rostral organization of the prefrontal cortex [31].

### Basic somatotopic organization

We used task-based data from N = 100 participant scanned in the Human Connectome Project [19] to provide a number of basic functional maps of the human cerebellum. The data set includes five motor conditions, involving simple movements of the left and right hand, left and right foot, and tongue. Fig 7a provides a composite map of the activation related to the movement of these body parts. Each vertex was assigned to the body part associated with the highest activation value. The image shows two ipsilateral representations of the body, one in the anterior cerebellum and the other (inverted) in the posterior areas. While the tongue-related activity was slightly weaker in the posterior lobe, especially on the right side, it is nonetheless visible in the un-thresholded maps. This topography largely corresponds to the sensorimotor organization reported previously [32]. Furthermore, the area of hand activation also corresponds well to the location of the regions that contain finger-specific information (Fig 1b, [17]). Finally, the somatotopy is also in line with the functional connectivity maps [18], where the more dorsal motor network (3) is co-located with activation during foot movements and the more ventral motor network (4) with activation during tongue movements.

## Activity maps from cognitive tasks

The remainder of the HCP task-based data set contains tasks that tap into various cognitive, emotional or social processes. While these data are described fully elsewhere [20], the original study did not report the cerebellar results in detail. Thus, in addition to demonstrating the use of the cerebellar flatmap in visualizing functional data, the analysis presented here contributes to the growing knowledge on the cerebellar involvement in cognitive and affective functioning.

Fig 7b shows the areas activated by the working memory task. The contrast presented here compares the 2-back and 0-back conditions. Significantly more activity in the 2-back task (*p*<0.05, FWE-corrected over the volume of the cerebellum) was found bilaterally in two areas of the most lateral parts of lobule HVI and HVII. This is broadly consistent with other studies showing load-dependent activity in working memory tasks [33–35].

In the emotion-processing task subjects were asked to match pairs of either emotional (angry or fearful) faces as compared to emotionally neutral shapes [20]. The most significant increase in BOLD signal related to the emotional content of the stimuli was observed in the right lateral lobule HVI (Fig 7c). Another large cluster extended from the vermis of the lobules VI/VII towards the hemispheres (Crus I and Crus II), primarily on the left side. These areas are again consistent with the regions implicated in emotional processing in a number of meta-analyses [33, 34] and functional studies [36–38]. Significant differences were additionally found bilaterally in lobule HX and the vermis of lobules IX/X. There is some evidence for the involvement of the vermal lobule IX in emotional processing [39]. The role of lobule HX, however, is unclear; lobule HX is associated mainly with vestibular functions [40], although some increased activity was observed in emotion processing when compared to the language domain [34].

Social cognition was measured with a theory of mind task in which subjects watched videos of moving shapes that either ‘interacted’ with each other or moved at random [20]. The comparison between social vs. random movement revealed significant cerebellar activation in three bilateral regions situated on the borders between lobule HVI/Crus I, Crus I/Crus II, as well as Crus II/ HVIIb (Fig 7d). These clusters are located more medial than the activation observed in the working-memory task, stressing the importance of the medial-lateral (as opposed to lobular) organization. This pattern of activity was confirmed by another imaging study using a similar experimental tasks [41]. Additionally, a large meta-analysis investigating the role of the cerebellum in social cognition implicated these areas in mentalizing tasks such as goal-directed motions [42].

The language domain was investigated by comparing story comprehension to a task in which participants had to perform arithmetic operations [20]. The story > math comparison (red-yellow, Fig 7e) revealed large areas in the bilateral posterior cerebellum, spanning Crus I and II. Smaller loci were also found in the most medial parts of HIX. The activity in Crus I confirms other studies using semantic tasks [43, 44] and also the results from meta-analyses [33, 34]. However these studies found strongly right-lateralized activations without corresponding increase in signal in the left hemisphere. While in the current data set [20] we still observe some degree of lateralization, the left cerebellum is also significantly activated.

The math > story contrast (Fig 7e, blue) showed higher activity for arithmetic operations in lateral as well as vermal areas of bilateral lobule HVI, Crus I and Crus II. The lateral areas largely overlap with the working memory network, revealed in the N-back task (Fig 7b), and therefore may reflect the involvement of executive processes.

The remaining two tasks in the functional data set [20] investigated relational processing (deciding which dimension differs across one set of stimuli, and whether this dimension also differs in the second set of stimuli) and reward processing. The contrast of relational decision-making against simple matching task did not show any significant clusters. The contrast of mostly-reward with mostly-punishment blocks in a card guessing game showed only two one- and two-voxel clusters in the vermis of lobule VII that just reached the significance threshold (not shown here).

In sum, the data shows that various cognitive, emotional and social mental processes activate the cerebellum in specific ways. The precise location of these activations can serve as a reference for further functional studies of the cerebellum.

## Discussion

Visualization methods are powerful and important scientific tools [45, 46]. The ability to “see” complex data in one figure exploits the human ability for visual pattern recognition, allows the researcher to relate so far unrelated observations to each other, form new connections, and to generate new hypotheses. They are also important in communicating scientific results efficiently, and provide a valuable teaching tool.

In most neuroimaging papers on the cerebellum the reader is shown a small number of slices, typically representing less than 10% of the volume. This prevents the appreciation of the full patterns of results—important aspects of the data can be overlooked, and the relationship between activation patterns can be misrepresented. Inflated or flattened surface-based representations of neuroimaging data are a powerful remedy for this limitation [1, 5] and are now widely adopted in many analysis pipelines for neocortical data [24].

Here we present a solution for visualizing cerebellar functional data on a 2D-surface. Representing resting-state networks and functional activation maps of motor and cognitive tasks on a flat representation relative to the lobular organization yielded some interesting, if not completely new, insights. One issue made very clear by this type of representation is that most cerebellar fissures do not correspond to functional boundaries—functionally defined regions usually span multiple lobules. Furthermore, the medial-to-lateral organization of the cerebellar cortex appears at least equally important as the anterior-to-posterior organization. From a physiological perspective this is to be expected; while the cerebellar cortex has a homogenous organization across the lobules, animal studies have shown that immunohistological staining can reveal a functional compartmentalization. In the medial-lateral direction, Aldolase-C positive and negative zones alternate [47, 48], which also reflect functional boundaries of climbing fiber input, nuclear projections of the Purkinje cells [49, 50], and of basic functional properties of Purkinje cells [51]. The practice to denote and summarize cerebellar neuroimaging data by the lobular compartment may have overemphasized the anterior-to-posterior at the expense of the medial-to-lateral organization. The use of the flatmap provides a simple method to view and summarize functional imaging data representing both directions equally.

A flat representation provides an effective and information-rich display of the precise localization and shape of foci of activation. This simple and readily interpretable visualization of task-related activity has a number of advantages compared to statistical tables, which are standard in the field of neuroimaging. First, the exact shape—the extension of activation cluster into neighboring regions—can be assessed, something that is virtually impossible when only the location of maxima and minima is reported. Second, it becomes apparent which areas are not activated by a task, which may be considered as informative as the presence of activation. Third, a surface-based representation of functional imaging results promotes the direct comparison with previous experiments—not only by assessing whether the same lobules were activated, but also by visually matching the entire pattern of activity. The data sets reported here [18, 20] provide such a reference. While it is preferable to compare the full functional maps between experiments, these are not always readily available. The toolbox therefore also contains functionality that lets the user plot single activation foci, defined by 3D-coordinates in atlas space, onto the flatmap. Finally, while we have focused here on the visualization of functional activation data—the flatmap can also be used to display anatomical data, for example from voxel-based morphometry [52] studies. Overall, the use of better visualization techniques should encourage a more thorough discussion of the functionally non-uniform structure of the cerebellum [33, 34, 53].

The accuracy of the mapping depends on the use of surfaces that match the volume-based normalization algorithm (see Fig 4). Furthermore, to prevent intrusion of functional signal from outside the cerebellum, it is advisable to map minimally smoothed data to the surface. Masking of the cerebellar volume before normalization and smoothing, as implemented in the SUIT toolbox [12], further decreases the danger of mis-localization of activity.

Because visualization techniques have a powerful influence on the understanding of data, it is important to appreciate the limitations of the suggested technique. Two-dimensional representations of 3D structures invariably introduce some distortions and overemphasize some areas at the expense of others. While the present flatmap indeed has some of these distortions, we attempted to make the surface area of each area roughly proportional to the corresponding grey-matter volume.

Furthermore, to flatten the 3D-surface it was necessary to make cuts to the surface, which renders contiguous regions non-contiguous (see Fig 3a). The user is therefore warned that even though lateral HVI and Crus I, the posterior vermis and the posterior hemispheres, and lobules HVIII and HX each appear to be separated on the flatmap, they are all directly adjacent in the volume. That means that functional activation can be mis-localized across these boundaries.

Naturally, the flatmap only displays data from the cerebellar gray matter and completely omits deeper structures. For functional data analysis, therefore, activation coming from the deep cerebellar nuclei needs to be analyzed and displayed using separate techniques [54]. While the omission of data from the cerebellar white matter is appropriate for BOLD activity data, it constitutes a severe limitation in the display of focal lesion data [55]. A lesion that extends into the cerebellar white matter may functionally disconnect large areas of the cerebellar hemispheres, but may look relatively small on the flatmap, because the disconnected cortical tissue is still preserved. Therefore, great care should be taken when using the current map for lesion analysis.

Finally, it is important to note that the current map is not a true unfolding of the cerebellar surface, but is based on surfaces reconstructed on group-averaged anatomical data. This contrasts dramatically with most surface-based reconstructions of neocortical data, where the cortical surface of each individual can be reliably reconstructed [1], which improves inter-subject alignment by accounting for variability in the cortical folding [5].

For the cerebellar map, our inability to reconstruct a surface for each participant means that each location on the flatmap averages over multiple folia. Thus, sampling data at different depth between the outer and inner surface may yield very different results. This implies—in contrast to the neocortex—that statistical analysis should still be performed in the space of the original 3D-data, rather than on the surface projection.

A true unfolding of the cerebellar cortex would yield a sheet of 700–1000 cm^2^, roughly equivalent to the size of a single cerebral hemisphere [10, 56]. Compared to the current flatmap, this sheet would have a similar width, but would be 1.5-2m long [10, 11] (resulting in a slightly awkward format for a journal figure). Such a representations would likely only make sense if the resolution of the functional data was around 1mm, and would necessitate an anatomical resolution ~200μm to reliably unfold the cerebellar volume [11]. Until the time that such resolutions become standard in functional imaging of the human cerebellum, the present map of volume-averaged data already provides some of the advantages in visualization of functional data, including increased power for exploratory data analysis [45] and more efficient communication of experimental results [46].
